# IL-6 Receptor Expression on the Surface of T Cells and Serum Soluble IL-6 Receptor Levels in Patients with Microscopic Polyangiitis and Granulomatosis with Polyangiitis

**DOI:** 10.3390/jcm12227059

**Published:** 2023-11-13

**Authors:** Taejun Yoon, Sung Soo Ahn, Eunhee Ko, Jason Jungsik Song, Yong-Beom Park, Sang-Won Lee

**Affiliations:** 1 BK21 Plus Project, Department of Medical Science, College of Medicine, Yonsei University, Seoul 03772, Republic of Korea; 2 Division of Rheumatology, Department of Internal Medicine, Yongin Severance Hospital, College of Medicine, Yonsei University, Yongin 16995, Republic of Korea; 3 Division of Rheumatology, Department of Internal Medicine, Severance Hospital, College of Medicine, Yonsei University, Seoul 03772, Republic of Korea; 4 Institute for Immunology and Immunological Diseases, College of Medicine, Yonsei University, Seoul 03772, Republic of Korea

**Keywords:** IL-6 receptor, activity, T cells, microscopic polyangiitis, granulomatosis with polyangiitis

## Abstract

We investigated the IL-6 receptor (IL-6R) expression on the surface of T cells isolated from peripheral blood mononuclear cells (PBMCs) of microscopic polyangiitis (MPA) and granulomatosis with polyangiitis (GPA) patients and measured the serum soluble IL-6R (sIL-6R) levels in these patients. Sera and PBMCs were obtained from 51 patients with MPA (*n* = 32) and GPA (*n* = 19), with 25 patients having active disease (defined as a Birmingham Vasculitis Activity Score [BVAS] ≥ 5). The median age of patients was 67.0 years, and 52.9% were women. Serum IL-6 levels were significantly correlated with the BVAS (r = 0.384); however, IL-6R expression on the surface of T cells did not significantly differ based on disease activity. Meanwhile, IL-6R expression on the surface of stimulated CD4+ (median mean fluorescence intensity [MFI] 588.0 vs. 1314.8; *p* < 0.001), CD4+CD25+ (MFI 853.3 vs. 1527.3; *p* < 0.001), and CD4+CD45RO+ (MFI 679.5 vs. 1241.5; *p* < 0.001) T cells was significantly reduced compared with unstimulated conditions. Conversely, patients with active disease exhibited a significantly higher median serum sIL-6R level than those with inactive disease (38.1 ng/mL vs. 34.7 ng/mL; *p* = 0.029). These results imply that the trans-signalling IL-6 pathway may be more activated than the classical signalling pathway in patients with MPA and GPA, suggesting the therapeutic potential of targeting sIL-6R.

## 1. Introduction

Microscopic polyangiitis (MPA) and granulomatosis with polyangiitis (GPA) are classified as antineutrophil cytoplasmic antibody (ANCA)-associated vasculitis (AAV), which is characterised by necrotising vasculitis with or without granuloma formation in small-sized vessels [1]. Among the various cytokines and chemokines, interleukin (IL)-6 plays diverse and critical roles in the pathogenesis of MPA and GPA, owing to the following reasons: First, IL-6 secreted from antigen-presenting cells along with transforming growth factor beta may drive T helper (Th)17 cell differentiation, which, in turn, may accelerate tumour necrosis factor alpha and IL-1β production, thereby inducing neutrophil priming [2,3,4]. Second, IL-6 may provoke monocyte activation, which may subsequently amplify the process of neutrophil activation and degranulation of lytic enzymes and reactive oxygen species, consequently augmenting endothelial and adjacent tissue inflammation [2,3,5]. Third, the induction of neutrophil extracellular trap (NET) formation (NETosis) could be mediated by IL-6, which may enhance ANCA production by an increased rate of myeloperoxidase (MPO) and proteinase 3 (PR3) release [5,6].

In general, the IL-6 intracellular signalling transduction pathway requires the generation of a membrane-bound glycoprotein 130 (gp130) dimer, which occurs after the formation of IL-6 and the IL-6 receptor (IL-6R) complex. The binding of IL-6 to IL-6R results in the IL-6R-linked gp130 dimer translocation to the nucleus, activating the Janus family tyrosine kinase (JAK) and signal transducers and activators of transcription (STAT) pathways to upregulate IL-6, IL-6R, and gp130 gene expression levels. This process is called the classical signalling pathway of IL-6 [7,8]. Conversely, in terms of soluble IL-6R (sIL-6R), when IL-6 binds to sIL-6R, IL-6 and the sIL-6R complex can be inserted between the two gp130s. The subsequent signalling process functions in almost the same way as the classical signalling pathway of IL-6. This process is known as the trans-signalling pathway of IL-6 [9,10]. However, one difference between the two pathways is the presence or absence of action by suppressors of cytokine signalling (SOCS). The expression of SOCS is regulated by IL-6 signalling, and while SOCS acts as a negative regulator of JAK-STAT signalling in the classical signalling pathway of IL-6, SOCS rarely plays its role in the trans-signalling pathway, indicating that these differences could influence the balance of inflammation in inflammatory disorders [8,11].

Previous studies reported that serum IL-6 levels in patients with MPA and GPA were elevated in comparison with those in healthy controls and were associated with cross-sectional AAV activity [12,13]. In addition, an increase in IL-6 expression in inflamed tissue from organs affected by AAV was reported [13]. Furthermore, based on the information on the role of IL-6 in the pathogenesis of AAV and the production and expression of IL-6 in the sera and tissues in these patients, several studies have been conducted on the use of tocilizumab, an inhibitory monoclonal antibody against both IL-6R and sIL-6R for disease treatment [13,14]. However, due to the lack of results with conclusive evidence from clinical trials, and owing to the insufficient number of patients, targeting the IL-6 pathway is not currently recommended in the guidelines for the management of MPA and GPA [15]. Additionally, the expression of IL-6R and soluble IL-6R levels, which are thought to exert important functions in promoting inflammation in MPA and GPA, has not been well understood in the existing literature.

In the present study, we evaluated the IL-6R expression in T cells and serum sIL-6R levels in patients with MPA and GPA, which could have potential implications for the clinical application of therapeutic IL-6 inhibition in MPA and GPA patients.

## 2. Materials and Methods

### 2.1. Patients and Healthy Controls

Fifty-one patients (32 MPA and 19 GPA), followed-up in a university-affiliated tertiary hospital, were included in the present study. All patients were classified as having MPA and GPA according to these four criteria: (i) the 2012 revised Chapel Hill Consensus Conference Nomenclature of Vasculitides [1]; (ii) the 2007 European Medicine Agency algorithm for AAV and polyarteritis nodosa [16]; (iii) the 2022 American College of Rheumatology (ACR)/European Alliance of Associations for Rheumatology (EULAR) classification criteria for MPA [17]; (iv) the 2022 ACR/EULAR classification criteria for GPA [18]. The medical records of the patients were sufficient to collect clinical, laboratory, histological, and radiological data when the sampling of blood was performed on the patients. At the time of blood sampling, the patients did not have any serious concomitant medical conditions other than MPA and GPA, such as severe infectious diseases or malignancies. A total of twenty-nine age- and sex-matched healthy subjects, consisting of 14 men and 15 women, served as healthy controls.

### 2.2. Data at the Time of Blood Sampling

Demographic data of the patients included age and sex, and the Birmingham Vasculitis Activity Score (BVAS) and the Korean version of the Short-Form 36-Item Health Survey Physical and Mental Component Summaries (SF-36 PCS and SF-36 MCS) were collected as disease-specific indices [19,20]. Data of medications that were being prescribed for disease treatment, as well as the information of organ involvement based on the subcategory of BVAS items, were obtained. For glucocorticoid usage, it was considered to be used in the patients when the daily dosage was equivalent to ≥5 mg of prednisone.

Laboratory results included MPO-ANCA (or perinuclear (P)-ANCA), PR3-ANCA (or cytoplasmic (C)-ANCA), white blood cell (WBC) and neutrophil counts, erythrocyte sedimentation rate (ESR), C-reactive protein (CRP), and serum creatinine. An immunoassay for MPO-ANCA and PR3-ANCA was used as the initial detection method for ANCA [21]. However, when patients had neither MPO-ANCA nor PR3-ANCA but had either P-ANCA or C-ANCA, they were also considered to be ANCA-positive [22].

### 2.3. Definition for Active and Inactive Disease

In the present study, we arbitrarily set a BVAS of 5 as the cut-off for active MPA and GPA, which could divide the patients evenly. Of the 51 patients, 25 and 26 belonged to the active (BVAS ≥ 5) and inactive (BVAS < 5) groups, respectively. In a separate analysis, a BVAS cut-off of ≥3 was applied to identify whether there is a difference in IL-6R expression according to the different BVAS cut-offs [23].

### 2.4. Isolation and Storage of Sera and Peripheral Blood Mononuclear Cells

Whole blood from patients who agreed to provide their samples was collected in a plain tube and an EDTA tube. Sera were immediately isolated from the plain tube samples and stored at −80 °C. Peripheral blood mononuclear cells (PBMCs) were isolated from the EDTA tube samples by Ficoll density-gradient centrifugation, stored at −80 °C in a freezing isopropanol container for 24 h, and transferred into the vapour phase of a liquid nitrogen tank.

### 2.5. Measurement of Serum IL-6 and sIL-6R Levels

Serum IL-6 and sIL-6R levels were measured from stored sera using the Human Magnetic Luminex^®^ assay (R&D Systems, Minneapolis, MN, USA) following the manufacturer’s instructions. Briefly, samples diluted 2-fold with commercial diluent were added to each well with the Microparticle Cocktail, and incubation was performed for 2 h at room temperature. Wells were washed 3 times, and the Biotin-Antibody Cocktail was added. Then, they were incubated for 1 h at room temperature. After 3 times washes, wells were incubated with Streptavidin-PE for 30 min at room temperature. A final wash was performed, and wells were read after 2 min of incubation with a commercial wash buffer using a Luminex^®^ MAGPIX instrument (Luminex, Austin, TX, USA). A 5-parameter logistic curve was used with standard optical density values for calculation.

### 2.6. Stimulation of PBMCs

Stored PBMCs from 10 patients with active disease were thawed rapidly and cultured in a 96 U-shaped well plate (SPL Life Sciences, Pocheon, Republic of Korea) in RPMI 1640 (Corning, NY, USA) supplemented with 10% foetal bovine serum (FBS, Corning, NY, USA), 100 U/mL penicillin, 100 μg/mL streptomycin, 1mM sodium pyruvate, and 20 mM HEPES, 0.1 mM non-essential amino acids, 20 μg/mL gentamycin, and 500 μM β-mercaptoethanol. Cells were then stimulated with phorbol 12-myristate 13-acetate (PMA) (50 ng/mL) and ionomycin (750 ng/mL) for 5 h.

### 2.7. Fluorescence-Activated Cell Sorting

Stored and stimulated PBMCs were gathered and washed with phosphate-buffered solution (PBS) supplemented with 1% FBS. Thereafter, cells were stained for 45 min with the following antibodies: anti-CD3-V500 and anti-CD8-V450 (BD Biosciences, Oxford, UK) and anti-CD4-Alexa Fluor 700, anti-CD25-APC, anti-CD45RO-PerCP-Cy5.5, and anti-CD126-PE (BioLegend, San Diego, CA, USA). Stained cells were washed with PBS supplemented with 1% FBS to remove residual free fluorochrome. Then, 2 × 10^5^ and 5 × 10^4^ gated lymphocytes were analysed for stored and stimulated PBMCs, respectively, using the FACSVerse and FlowJo v10 software (BD Biosciences, Oxford, UK).

### 2.8. Statistical Analyses

All statistical analyses were performed using IBM SPSS Statistics for Windows, version 26 (IBM Corp., Armonk, NY, USA). Continuous variables were expressed as medians with interquartile ranges, whereas categorical variables were expressed as numbers (percentages). Significant differences between the two categorical variables were analysed using the Chi-square and Fisher’s exact tests as appropriate. Significant differences between two continuous variables were compared using the Mann–Whitney U test. The correlation coefficient (r) between the two variables was obtained using the Pearson correlation analysis. A value of two-tailed *p* < 0.05 was considered statistically significant.

## 3. Results

### 3.1. Patients’ Characteristics

The median age of the 51 patients was 67.0 years, and 52.9% of them were women. The median BVAS, SF-36 PCS, and SF-36 MCS were 4.0, 66.6, and 62.5, respectively. Among the BVAS items, the most frequently affected organ was the kidney (56.9%), followed by the lungs (43.1%) and the ear, nose, and throat (33.3%). MPO-ANCA (or P-ANCA) and PR3-ANCA (or C-ANCA) were detected in 62.7% and 7.8% of the patients, respectively, and 15 patients were negative for ANCA (29.4%). The median ESR and CRP were 15.0 mm/h and 2.1 mg/L, respectively (Table 1).

### 3.2. Comparison of Characteristics between Patients with Active and Inactive Disease

There were no differences in the demographic data among patients with active and inactive disease; however, patients with active disease exhibited lower SF-36 PCS (57.5 vs. 67.0, *p* = 0.045) and MCS (58.8 vs. 70.3, *p* = 0.008) than those with inactive disease. The proportion of patients on azathioprine was higher in the inactive disease group (*p* = 0.050).

Among the BVAS items, patients that belonged to the active disease group had significantly higher frequencies of general (24.0% vs. 0%; *p* = 0.010), pulmonary (64.0% vs. 23.1%; *p* = 0.004), and nervous systemic manifestations (44.0% vs. 15.4%; *p* = 0.034). For the laboratory results, patients with active disease had a lower detection rate of MPO-ANCA (or P-ANCA) compared with those with inactive disease (*p* = 0.007). Additionally, compared with patients with inactive disease, those in the active disease group had elevated counts of white blood cells (8420.0/mm^3^ vs. 6310.0/m^3^; *p* = 0.012) and neutrophils (6300.0/mm^3^ vs. 3700.0/mm^3^; *p* = 0.007). However, there were no significant differences in ESR, CRP, and serum creatinine levels between the two groups (Table 1).

### 3.3. Correlation of Serum IL-6 Levels with Disease-Specific Indices and Acute-Phase Reactants

**Figure 1:**
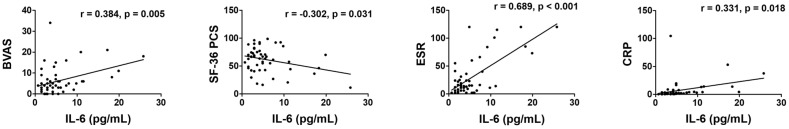
Serum IL-6 levels showed a significant correlation with BVAS, SF-36 PCS, ESR, and CRP. IL-6: interleukin-6; BVAS: Birmingham Vasculitis Activity Score; SF-36: Short-Form 36-Item Health Survey; PCS: physical component summary; ESR: erythrocyte sedimentation rate; CRP: C-reactive protein.

In a correlation analysis, it was demonstrated that serum IL-6 levels were significantly correlated with cross-sectional BVAS (r = 0.384; *p* = 0.005), SF-36 PCS (r = −0.302; *p* = 0.031), ESR (r = 0.689; *p* < 0.001), and CRP (r = 0.331; *p* = 0.018) (Figure 1).

### 3.4. Comparison of Serum IL-6 Levels between the Groups

**Figure 2:**
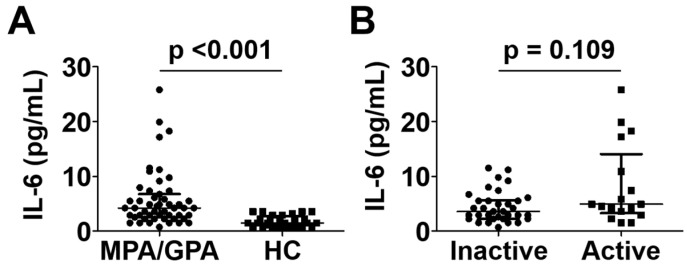
Comparison of serum IL-6 levels between MPA and GPA patients and healthy controls (**A**) and between patients with active disease and inactive disease (**B**). Data are presented as median with interquartile range. IL-6: interleukin-6; MPA: microscopic polyangiitis; GPA: granulomatosis with polyangiitis; HC: healthy control.

Patients with MPA and GPA exhibited a significantly higher median serum IL-6 level than controls (4.2 pg/mL vs. 1.5 pg/mL; *p* < 0.001) (Figure 2A), which was consistent with the results of previous studies [12,13]. On the other hand, serum IL-6 levels in patients with active disease were not significantly elevated compared to those in patients with inactive disease (4.9 pg/mL vs. 3.6 pg/mL; *p* = 0.109) (Figure 2B).

### 3.5. Comparison of IL-6R Expression on the Surface of T Cells between Patients with Active and Inactive Disease

**Figure 3:**
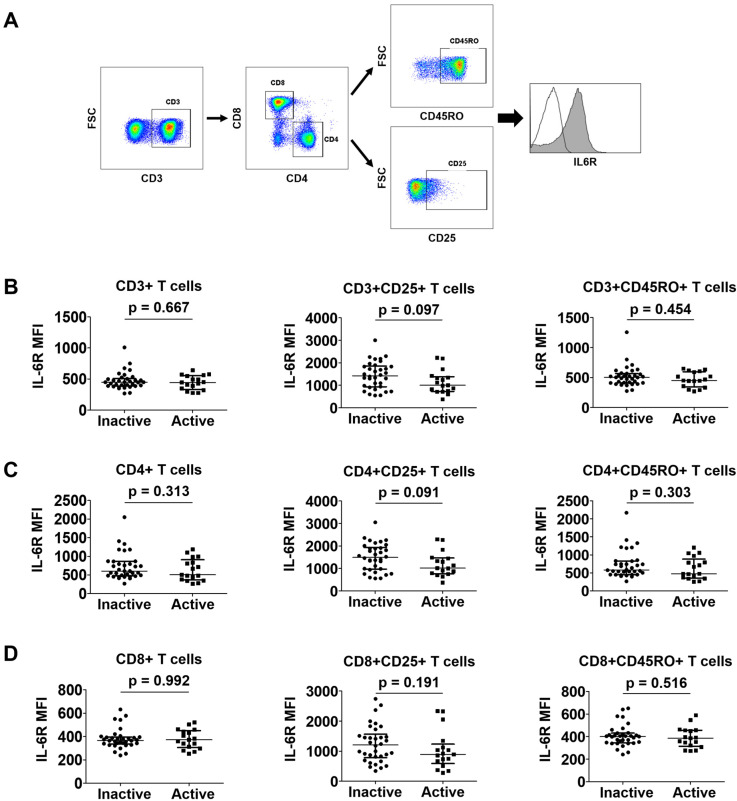
Comparison of IL-6R expression in T cells between patients with active disease and those with inactive disease. A representative image described the gating strategy (**A**). Comparison of CD3+ (**B**), CD4+ (**C**), and CD8+ (**D**) T cell subsets in patients in the active and inactive disease groups. Data are presented as median with interquartile range. IL-6R: interleukin-6 receptor; FSC: forward scatter; MFI: mean fluorescence intensity.

A gating strategy for evaluating the IL-6R expression on CD3+, CD3+CD4+, and CD3+CD8+ T cells is presented in Figure 3A. CD25+ T cells represent an activated state, and CD45RO+ cells represent memory T cells (Figure 3A). In terms of CD3+ T cells, patients with active disease showed a tendency towards decreased IL-6R expression on the surface of CD3+CD25+ T cells compared with those with inactive disease; however, this was not statistically significant (median mean fluorescence intensity [MFI] 1000.0 vs. 1425.0; *p* = 0.097). Conversely, no significant differences in IL-6R expression on the surface of CD3+ or CD3+CD45RO+ T cells were observed between the two groups (Figure 3B). In terms of CD4+ T cells, IL-6R expression on the surface of CD4+CD25+ T cells in patients with active disease tended to be lower than that in patients with inactive disease (MFI 1016.0 vs. 1490.0; *p* = 0.091), but this also did not reach statistical significance. Similarly, there were no differences in IL-6R expression on the surface of CD4+ or CD4+CD45RO+ T cells between the two groups, identical to that found for the CD3+ T cells (Figure 3C). Finally, regarding CD8+ T cells, IL-6R expression on the surface of CD8+, CD8+CD25+, or CD8+CD45RO+ T cells exhibited no significant differences between patients with active and inactive disease (Figure 3D). These patterns were revealed to be identical even when a different cut-off of BVAS (BVAS ≥ 3) was applied to define active disease and inactive disease [23] (Supplementary Figure S1).

### 3.6. Comparison of Surface IL-6R Expression between Stimulated and Unstimulated T Cells

**Figure 4:**
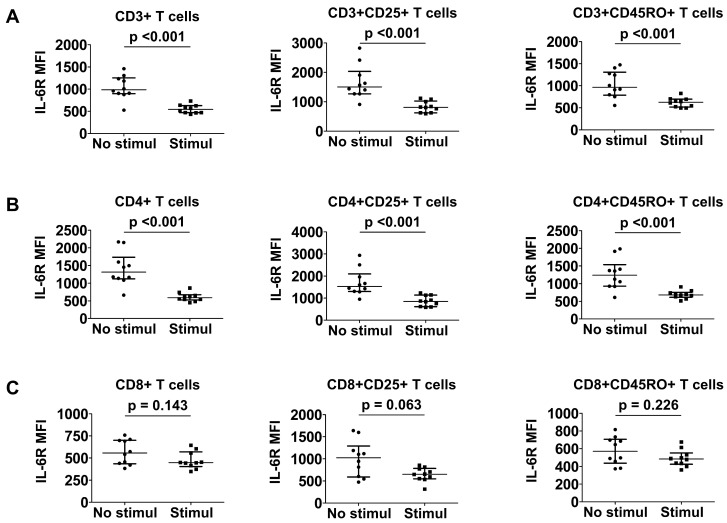
Comparison of IL-6R expression between stimulated and unstimulated T cells. CD3+ (**A**), CD4+ (**B**), and CD8+ (**C**) T cell subsets before and after stimulation. Data are presented as median with interquartile range. IL-6R: interleukin-6 receptor; MFI: mean fluorescence intensity; Stimul: stimulation.

Since the stimulation of T cells could result in a differential effect of IL-6R expression in patients with MPA and GPA, surface IL-6R expression in T cells was compared before and after T cell stimulation. Regarding CD3+ T cells, IL-6R expression on the surface of stimulated CD3+ (MFI 543.5 vs. 985.5; *p* < 0.001), CD3+CD25+ (MFI 811.3 vs. 1502.8; *p* < 0.001), and CD3+CD45RO+ (MFI 626.5 vs. 964.3; *p* < 0.001) T cells was significantly reduced in each of these groups compared with unstimulated T cells (Figure 4A). Similarly, T cell stimulation resulted in a significant decrease in the expression of IL-6R on the surface of stimulated CD4+ (588.0 vs. 1314.8, *p* < 0.001), CD4+CD25+ (853.3 vs. 1527.3, *p* < 0.001), and CD4+CD45RO+ (679.5 vs. 1241.5, *p* < 0.001) T cells (Figure 4B). In contrast, IL-6R expression on the surface of stimulated CD8+, CD8+CD25+, or CD8+CD45RO+ T cells did not appear to differ from that of unstimulated T cells (Figure 4C).

### 3.7. Comparison of Serum sIL-6R Levels between the Two Groups

**Figure 5:**
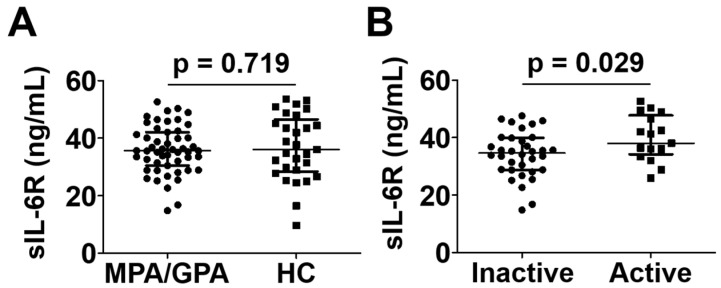
Serum sIL-6R levels in patients with MPA and GPA and healthy controls (**A**) and in those with active and inactive disease (**B**). Data are presented as median with interquartile range. sIL-6R: soluble interleukin-6 receptor; MPA: microscopic polyangiitis; GPA: granulomatosis with polyangiitis; HC: healthy control.

While the level of sIL-6R level was comparable in patients with MPA and GPA to controls, patients with active MPA and GPA exhibited a significantly higher median serum sIL-6R level than those with inactive disease (38.1 ng/mL vs. 34.7 ng/mL; *p* = 0.029) (Figure 5).

## 4. Discussion

In the present study, we investigated the alterations in IL-6R expression on the cell surface of T cells and serum sIL-6 levels according to disease activity or before and after stimulation and obtained several interesting results. First, patients with MPA and GPA had significantly higher serum IL-6 levels compared with controls, and those with active disease tended to have elevated serum IL-6 levels compared with those with inactive disease, although statistical significance was not obtained. Next, serum IL-6 levels were significantly correlated with the BVAS in patients with MPA and GPA; furthermore, sIL-6R levels were found to be higher in patients with active disease compared with the inactive disease group. These results suggest that the IL-6 signalling pathway plays an important role in modulating the inflammatory cascade in MPA and GPA, and intervening this pathway may have benefits in reducing disease severity.

Our observations revealed that IL-6R expression on the surface of CD3+CD25+ and CD4+CD25+ T cells in patients with active disease tended to be reduced compared with those with inactive disease; notably, IL-6R expression on the surface of stimulated CD3+ and CD4+ T cells was significantly lower than that of unstimulated T cells. This suggests that IL-6R might be downregulated by high disease activity associated with the IL-6 signalling pathway. Conversely, serum sIL-6R levels in patients with active disease were significantly higher than those in patients with inactive disease. This implies that sIL-6R, which is produced by the cleavage of IL-6R or released endogenously, might be upregulated by hyper-inflammation associated with the IL-6 signalling pathway. Therefore, it could be hypothesised that sIL-6R, rather than IL-6R, may be the main contributor to the pathogenesis of MPA and GPA at the active state level.

**Figure 6:**
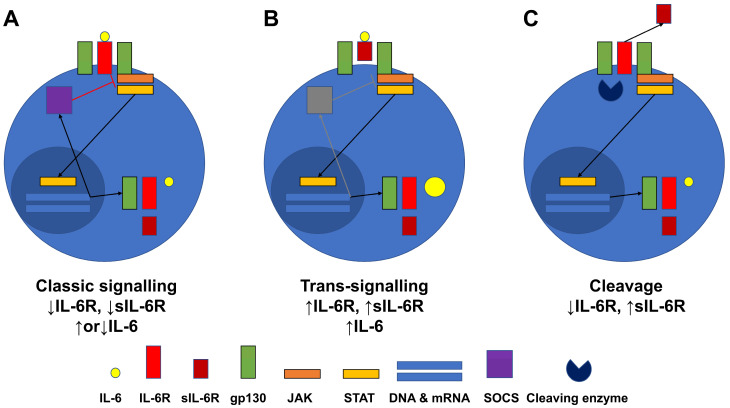
Hypothesis regarding the alteration in IL-6R expression and sIL-6 levels in MPA and GPA patients. The classic signalling (**A**) and trans-signalling (**B**) pathways, as well as cleaved sIL-6R (**C**), could promote alterations in IL-6R expression and sIL-6 levels. IL-6R: interleukin-6 receptor; sIL-6R: soluble interleukin-6 receptor; MPA: microscopic polyangiitis; GPA: granulomatosis with polyangiitis; gp130: glycoprotein 130; JAK: Janus family tyrosine kinase; STAT: signal transducers and activators of transcription; DNA: deoxyribose nucleic acid; mRNA: messenger ribonucleic acid; SOCS: suppressors of cytokine signalling.

Based on the results obtained herein, we hypothesise that IL-6R expression was reduced in stimulated CD4+ T cells and that serum sIL-6R levels were elevated in patients with active AAV for the following reasons: First, both IL-6R and sIL-6R production may be negatively regulated by the classical signalling pathway upon stimulation. Once IL-6 binds to IL-6R, IL-6R interacts with gp130, which forms a dimer and activates the JAK-STAT pathway. Nuclear translocated STAT may augment the expression of IL-6, IL-6R, sIL-6R, and gp130, which could also enhance the expression of SOCS, which inhibits the JAK signalling, consequently resulting in the downregulation of IL-6R and sIL-6R expression [24] (Figure 6A). Second, IL-6R and sIL-6R production may both be enhanced by the trans-signalling pathway in patients with active disease. Once IL-6 binds to sIL-6R, sIL-6R is inserted into the dimer of gp130, which activates the JAK-STAT pathway, resulting in the augmented expression of IL-6, IL-6R, sIL-6R, and gp130 in a similar manner to the classical signalling pathway. However, there might not be an apparent increase in SOCS expression compared with that observed in the classical signalling pathway, which could be related to this finding [25] (Figure 6B). The cleavage of IL-6R to sIL-6R may downregulate IL-6R expression in CD4+ T cells, concomitantly upregulating released serum sIL-6R levels (Figure 6B). Subsequently, cleaved sIL-6R could have two fates. One is that sIL-6R may couple with gp130 dimers and participate in the trans-signalling pathway, contributing to aggravated inflammation by amplifying the production of inflammatory cytokines (Figure 6B). The other is that cleaved sIL-6R might couple with soluble gp130 dimers and inhibit both signalling pathways competitively, resulting in the diminished expression of STAT-transcribing downstream genes, particularly of inflammatory cytokines [26,27] (Figure 6C). Nonetheless, as the total amount of sIL-6R increases, sIL-6R may be more capable of accelerating the trans-signalling pathway and promoting inflammation.

In the third hypothesis, the role of disintegrin and metalloproteinase (ADAM) 10 and 17, which are primary enzymes involved in the cleavage of IL-6R to sIL-6R, is crucial in upregulating sIL-6R levels. This is because the increased expression and function of ADAM 10 and 17 proteins are closely related to the exacerbation of inflammation by upregulating sIL-6R levels and accelerate the activation of the IL-6-related trans-signalling pathway. A previous study also demonstrated that a high inflammatory burden augmented the expression of ADAM 10 and 17 in patients with AAV [28]. These findings might support the results of our experiment that evaluates sIL-6R expression in patients with active disease.

In the present study, there were different patterns of IL-6R expression on the surface of simulated CD4+ and CD8+ T cells. Stimulated CD4+ T cells showed significantly reduced IL-6R expression compared with unstimulated T cells, whereas CD8+ T cells did not show a similar pattern to CD4+ T cells. It is assumed that these results are attributable to the use of T cells obtained from PBMCs rather than from inflamed tissue. The role of effector or cytotoxic T cells is also important in inflamed tissues; however, the main immune cells involved in the pathogenesis of AAV are B, Th1, and Th17 cells [29]. Therefore, our results that IL-6R expression was more evident in CD4+ T cells than in CD8+ T cells appear to be consistent with the previous findings.

Focusing on IL-6 expression on the surface of CD4+ T cells, although PMA and ionomycin stimulation caused a significant decrease in IL-6R expression, disease activity (active vs. inactive) only showed a trend towards reduced IL-6R expression, without statistical significance. Of the two results, we have more confidence in the results of the experiment using T cells stimulated with PMA and ionomycin. This is because IL-6R expression on the surface of T cells in active disease and inactive disease may be a complicated result due to various types of regulation and feedback, whereas IL-6R expression on the surface of T cells after direct stimulation may reflect a more direct response to microenvironmental immunity.

The present study is unique in that it is the first to demonstrate alterations in IL-6R expression on the surface of T cells as well as sIL-6R levels in patients with MPA and GPA, which could provide certain evidence of IL-6 suppression for optimal disease treatment. In addition, to minimise the confounding factors, patients with eosinophilic granulomatosis with polyangiitis (EGPA) were not included as a subtype of AAV, as the pathogenesis of EGPA is somewhat different from that of MPA and GPA, particularly regarding T cell subsets [30]. However, the present study has several limitations. First, it was designed as a pilot study. This could have led to a potential selection bias, and the number of patients may not have been large enough to reach statistical significance. Moreover, the arbitrary cut-off of BVAS of over 5 to define active disease could have influenced the study results. Second, the present study did not measure IL-6 and sIL-6R in the supernatant of treated T cells or IL-6R expression by cell sorting in controls. Third, alterations in the population of CD4+ T cell subsets such as Th1, Th2, Th17, and Treg cells were not investigated here. Fourth, the present study had a relatively high proportion (nearly 30%) of patients that were negative for ANCA. Finally, the alteration in IL-6R expression on the surface of T cells that had infiltrated into inflamed tissues could not be evaluated. A future study including more patients and addressing these limitations will provide more reliable evidence for differing IL-6R expression on the surface of distinct types of T cells and justify the utilization of IL-6 inhibitors for the treatment of MPA and GPA.

In conclusion, the findings of the present study demonstrated that IL-6R expression is decreased on the surface of stimulated CD3+ and CD4+ T cells from patients with MPA and GPA; in addition, serum sIL-6R levels in patients with active disease were significantly higher than those in patients with inactive disease. Our results imply that in the peripheral blood circulation, the trans-signalling IL-6 pathway may be more activated than the classical IL-6 signalling pathway in patients with MPA and GPA. To confirm these results, an additional study with a larger number of patients and stricter selection criteria remains essential to better differentiate active from inactive forms of vasculitis.

## Figures and Tables

**Table 1:** Patients’ characteristics and comparison between patients with active disease and those with inactive disease.

Variables	Total Patients (*n* = 51)	Active (*n* = 25) (BVAS ≥ 5)	Inactive (*n* = 26) (BVAS < 5)	*p*-Value
Patient demographics				
Age (years)	67.0 (51.5–74.0)	65.0 (50.8–71.3)	69.0 (55.0–76.0)	0.309
Female sex, *n* (%)	27 (52.9)	12 (48.0)	15 (57.7)	0.493
AAV subtypes				0.694
MPA, *n* (%)	32 (62.7)	15 (60.0)	17 (65.4)	
GPA, *n* (%)	19 (37.3)	10 (40.0)	9 (34.6)	
Disease-specific indices				
BVAS	4.0 (2.0–7.8)	8.0 (6.0–15.3)	2.0 (0.0–3.0)	<0.001
SF-36 PCS	66.6 (46.3–74.8)	57.5 (34.5–70.2)	67.0 (56.6–88.8)	0.045
SF-36 MCS	62.5 (45.5–75.3)	58.8 (36.0–64.4)	70.3 (50.3–85.1)	0.008
Current medication usage, *n* (%)				
Glucocorticoid	40 (78.4)	21 (84.0)	19 (73.1)	0.499
Cyclophosphamide	4 (7.8)	4 (16.0)	0 (0.0)	0.051
Rituximab	1 (2.0)	1 (4.0)	0 (0.0)	0.490
Azathioprine	17 (33.3)	5 (20.0)	12 (46.2)	0.050
Methotrexate	2 (3.9)	1 (4.0)	1 (3.8)	0.999
Organ involvement, *n* (%)				
General	6 (11.8)	6 (24.0)	0 (0.0)	0.010
Cutaneous	2 (3.9)	2 (8.0)	0 (0.0)	0.235
Mucous membrane/eyes	3 (5.9)	3 (12.0)	0 (0.0)	0.110
ENT	17 (33.3)	11 (44.0)	6 (23.1)	0.117
Pulmonary	22 (43.1)	16 (64.0)	6 (23.1)	0.004
Cardiovascular	4 (7.8)	4 (16.0)	0 (0.0)	0.051
Gastrointestinal	0 (0.0)	0 (0.0)	0 (0.0)	0.999
Renal	29 (56.9)	17 (68.0)	12 (46.2)	0.119
Nervous systemic	15 (29.4)	11 (44.0)	4 (15.4)	0.034
Laboratory results				
MPO-ANCA (or P-ANCA) positivity, *n* (%)	32 (62.7)	11 (44.0)	21 (80.8)	0.007
PR3-ANCA (or C-ANCA) positivity, *n* (%)	4 (7.8)	4 (16.0)	0 (0.0)	0.051
ANCA-negative, *n* (%)	15 (29.4)	10 (40.0)	5 (19.2)	0.107
WBC count (/mm^3^)	7570.0 (5940.0–9280.0)	8420.0 (6230.0–10,720.0)	6310.0 (5890.0–7850.0)	0.012
Neutrophil count (/mm^3^)	4700.0 (3300.0–6600.0)	6300.0 (4000.0–7600.0)	3700.0 (3200.0–5700.0)	0.007
ESR (mm/h)	15.0 (6.5–39.0)	23.0 (8.3–76.0)	15.0 (6.0–22.0)	0.095
CRP (mg/L)	2.1 (0.8–4.2)	3.0 (1.1–13.9)	1.8 (0.8–3.3)	0.092
Serum creatinine (mg/dL)	1.1 (0.8–1.7)	1.3 (0.8–2.5)	1.1 (0.8–1.5)	0.283

Values are expressed as the median with the interquartile range or number (percentage). BVAS: Birmingham Vasculitis Activity Score; AAV: ANCA-associated vasculitis; ANCA: antineutrophil cytoplasmic antibody; MPA: microscopic polyangiitis; GPA: granulomatosis with polyangiitis; SF-36: Short-Form 36-Item Health Survey: PCS: physical component summary; MCS: mental component summary; ENT: ear, nose, and throat; MPO: myeloperoxidase; P: perinuclear; PR3: proteinase 3; C: cytoplasmic; WBC: white blood cell; ESR: erythrocyte sedimentation rate; CRP: C-reactive protein.

## Data Availability

The data are available from the corresponding author on reasonable request.

## Supplementary Materials

The following supporting information can be downloaded at: https://www.mdpi.com/article/10.3390/jcm12227059/s1.
