# Supplementary material for: IL-6 Receptor Expression on the Surface of T Cells and Serum Soluble IL-6 Receptor Levels in Patients with Microscopic Polyangiitis and Granulomatosis with Polyangiitis
Source: J Clin Med. 2023 Nov 13;12(22):7059. doi: 10.3390/jcm12227059 (PMC10672709; doi:10.3390/jcm12227059)

Supplementary Figure S1. IL-6R expression in T cells between patients with active disease and those with inactive disease according to the BVAS cut-off of 3. Comparison of CD3+ (A), CD4+ (B), and CD8+ (C) T cells subsets among the two groups. IL-6R: interleukin-6 receptor; BVAS: Birmingham Vasculitis Activity Score; MFI: mean fluorescence intensity.

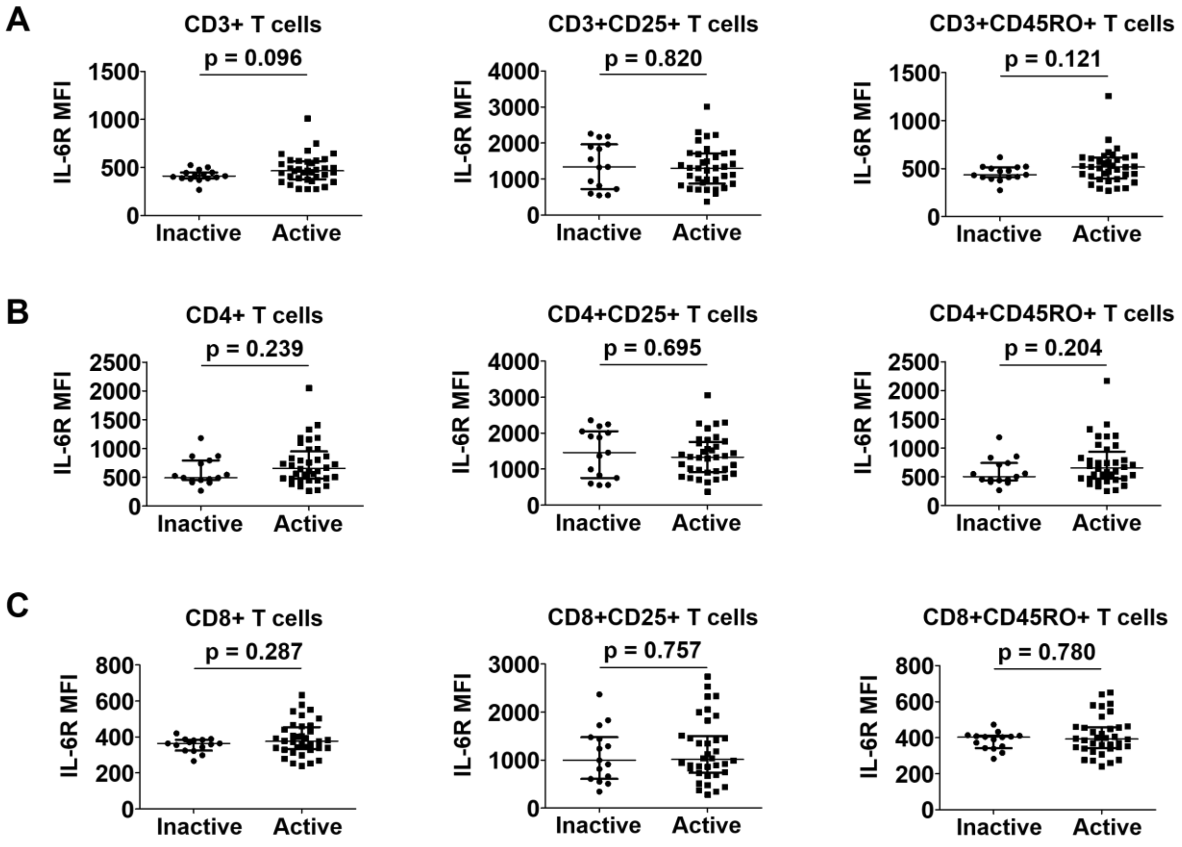

Supplement: Supplementary file 1 [file jcm-12-07059-s001.zip › jcm-2617999-supplementary.pdf]
